# Low-Substitution Glycerol Etherification of Guar Gum for Reduced-Residue Fracturing Fluids

**DOI:** 10.3390/gels12070619

**Published:** 2026-07-09

**Authors:** Yongfei Li, Boyang Shen, Rong Li, Huili He, Shiyu Wang, Qian Wang, Maogang Li, Gang Chen

**Affiliations:** 1 Engineering Research Center of Oil and Gas Field Chemistry, Universities of Shaanxi Province, Xi’an Shiyou University, Xi’an 710065, Chinalmglmg1995@163.com (M.L.); 2 Shaanxi Province Key Laboratory of Environmental Pollution Control and Reservoir Protection Technology of Oilfields, Xi’an Shiyou University, Xi’an 710065, China; 3 Karamay Sanda High-Tech Co., Ltd., Karamay 834000, China

**Keywords:** guar gum, glycerol etherification, fracturing fluid, rheology, insoluble residue, structure–property relationship

## Abstract

Reducing water-insoluble residues while maintaining sufficient rheological performance remains a key challenge for guar gum-based fracturing fluid thickeners. Natural guar gum (GG) is widely used in fracturing fluids, but its relatively high content of water-insoluble residues can impair permeability and reduce fracture conductivity. In this study, GG was modified by low-substitution etherification using a glycerol ether-based modifier (GMH-1) under mild alkaline reaction conditions to develop a reduced-residue thickener for fracturing fluid applications. The modification conditions were optimized through an L9 orthogonal design combined with single-factor analysis. Under the optimal conditions of 35 °C, 2.0 h, 2.0 wt % NaOH, and 0.2 wt % GMH-1, the modified product (GMGG) exhibited an apparent viscosity of 125.3 mPa·s and a water-insoluble residue content of 5.1% in a 0.6 wt % aqueous solution; in comparison, GG showed a viscosity of 89.3 mPa·s and a residue content of 12.4%. FTIR, UV-Vis spectroscopy, and elemental analysis provided indirect but consistent evidence for low-substitution chemical modification and the introduction of oxygen-containing hydrophilic groups while largely preserving the polysaccharide backbone. GMGG also showed improved rheological and thermal response behavior, suggesting that low-substitution glycerol etherification may provide a feasible route to balance residue reduction and viscosity enhancement. These results indicate the potential of this strategy for designing reduced-residue guar-based thickeners for fracturing fluids, while further molecular-level characterization is still required to determine the exact substitution pattern and mechanism.

## 1. Introduction

Hydraulic fracturing is a key stimulation technology to improve the productivity of unconventional oil and gas reservoirs. The overall performance of the fracturing fluid system largely depends on polymer thickeners, which are expected to provide sufficient viscosity for proppant transport, maintain rheological stability under downhole temperature and shear conditions, exhibit controllable gel-breaking behavior, and minimize residue-induced formation damage [1,2]. Equally importantly, excessive solid residues may be retained in pore throats and proppant packs after gel breaking and flowback, thereby reducing fracture conductivity and causing irreversible reservoir damage [3,4].

**Figure 1:**
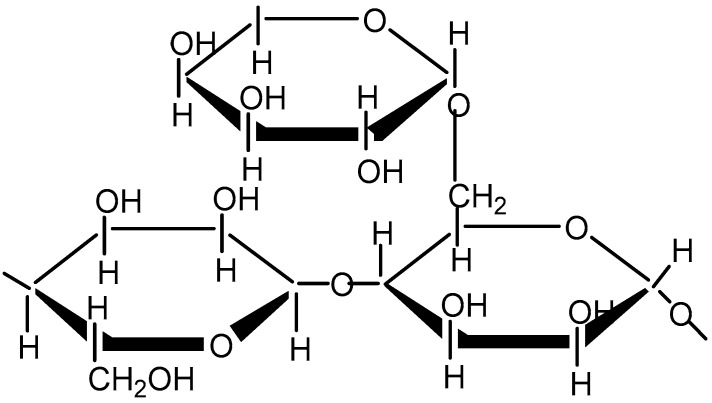
Chemical structure of the repeating unit of GG.

Guar gum (GG) and its derivatives are widely used in fracturing fluid systems due to their excellent thickening efficiency, renewability and good cost performance. However, natural GG usually contains about 10–14 wt % water-insoluble residues, mainly derived from cell wall fragments, residual proteins and some insoluble galactomannan structures, as shown in Figure 1 [5]. During fracturing construction and subsequent flowback, these insoluble components may be retained in fracture networks and near-wellbore zones, leading to a decrease in permeability. Therefore, reducing the content of water-insoluble residues while maintaining sufficient rheological properties has remained a key technical challenge for GG-based fracturing fluids [3,6].

In response to the above problems, researchers have proposed a variety of chemical modification methods for GG, including hydroxypropylation, carboxymethylation, sulfonation and cationization [7]. Among them, hydroxypropyl guar gum (HPG) has become a widely used standard thickener in the industry due to its improved hydration behavior and temperature resistance [2,8]. In practical fracturing applications, a standard thickener is generally expected to hydrate rapidly, provide sufficient base-fluid viscosity, form a stable crosslinked network, tolerate temperature and shear, break cleanly after treatment, and generate low residue and low formation damage. However, traditional hydroxypropylation usually relies on volatile and potentially toxic reagents such as propylene oxide, and commercial HPG may still generate residues under high-temperature or ultra-tight reservoir conditions. Although carboxymethylation and sulfonation can improve hydrophilicity, they often require a relatively high degree of substitution, which may lead to decreased rheological stability or increased cost. Cationic GG can inhibit clay swelling to some extent, but its use in fracturing fluids is still limited by system compatibility and economy [9].

In recent years, research on the etherification modification of GG using epoxy-based reagents (such as glycerol ether and its derivatives) has gradually attracted attention [10]. Existing studies have shown that the introduction of hydrophilic ether side chains helps to improve the hydration behavior of polymers and reduce the content of water-insoluble residues to a certain extent. However, most relevant studies rely on a high degree of substitution or strong reaction conditions to achieve performance improvement, which may lead to excessive structural modification, increased material cost and decreased rheological stability. In addition, existing studies often fail to clearly distinguish the respective contributions of chemical graft modification and post-treatment physical purification steps (such as solvent washing) to performance improvement, thus increasing the uncertainty in understanding the structure-property relationship [11].

Therefore, there are still two key issues to be clarified in existing studies: first, whether effective residue reduction and acceptable rheological performance can be achieved under mild reaction conditions and a low modification degree without excessive alteration of the GG backbone; second, how the respective roles of chemical etherification and post-treatment physical purification steps, such as solvent washing, can be distinguished when evaluating residue reduction and performance improvement. In this work, the phrase ‘mild reaction conditions’ refers to the etherification synthesis conditions, rather than to the high-temperature and high-pressure environment encountered during industrial fracturing operations.

Building on the aforementioned background, this study proposes a low-substitution etherification strategy for GG using a glycerol ether modifier (GMH-1) under mild alkaline reaction conditions. Distinct from traditional hydroxypropylation, the glycerol ether side chain introduced in this work contains additional hydroxyl groups that may enhance hydration and dispersion. We hypothesize that effective residue reduction may not necessarily require a high degree of substitution. Instead, limited introduction of hydrophilic ether side chains may help balance improved hydration, residue control, and rheological stability. This proposed structure-property relationship provides the basis for subsequent evaluation, but the exact substitution pattern and molecular mechanism require further verification by more direct structural methods [12].

Accordingly, the specific objectives of this study are to: (1) optimize the mild etherification process of GG with GMH-1 via orthogonal experimental design; (2) characterize the structural features of the modified product (GMGG) and distinguish the contribution of chemical modification from that of physical purification using a blank control; (3) evaluate the thermal response, rheological behavior, and engineering properties of GMGG, with commercial HPG as a comparative reference; and (4) discuss a plausible structure-property relationship responsible for its low-residue characteristics without overinterpreting indirect structural evidence [13].

## 2. Results and Discussion

### 2.1. Orthogonal Experimental Results

Based on the L9(3^4^) orthogonal experimental design described in Section 4.3, a total of nine etherification reactions were completed to systematically investigate the effects of GMH-1 dosage, NaOH dosage, reaction time, and reaction temperature on the modification effect [14]. All nine products were analyzed for apparent viscosity and water-insoluble residue content. The optimized sample was then selected for more detailed structural, rheological, and engineering performance evaluation. The apparent viscosity and water-insoluble residue content were normalized according to the comprehensive evaluation method, and the comprehensive evaluation index was calculated. The results are shown in Table 1.

Taking the comprehensive evaluation index as the response variable, range analysis was carried out to obtain the average values (K_1_, K_2_, K_3_) and range (R) of each factor at different levels. The results are shown in Table 2.

It can be seen from Table 2 that the range value of GMH-1 dosage (D) is the largest, followed by NaOH dosage (C), reaction time (B), and reaction temperature (A). Therefore, the apparent order of influence based on range analysis is D > C > B > A. It should be noted that the L9(3^4^) design is mainly suitable for screening the main effects of the four factors, and the interaction effects among factors were not explicitly resolved in this design. Potential interactions, especially between NaOH dosage and GMH-1 dosage, should therefore be examined by response surface methodology or a full-factorial design in future work.

Combined with the K values, the optimal reaction parameters were D_2_C_2_B_3_A_2_, namely GMH-1 dosage 0.20%, NaOH dosage 2.0%, reaction time 2.0 h, and reaction temperature 35 °C. This combination was selected because it provided the best overall balance between viscosity enhancement and residue reduction within the investigated experimental range.

Equal weighting factors were assigned to apparent viscosity and insoluble residue content because the optimization aimed to balance thickening performance with residue reduction. Excessive emphasis on viscosity may increase the risk of residue-induced formation damage, whereas excessive emphasis on residue reduction may compromise proppant-carrying capacity. Therefore, the equal-weighting scheme was adopted as a balanced criterion for preliminary optimization. In practical applications, the weighting factors can be adjusted according to reservoir sensitivity and fracturing design requirements.

To further evaluate the statistical reliability of the optimization results, ANOVA was performed using the comprehensive evaluation index as the response variable. The results are shown in Table 3. Because the L9 design does not provide pure error degrees of freedom, factor A, which showed the smallest contribution in the range analysis, was pooled into the error term for estimating the mean square error. This approach is commonly used as an approximate analysis for orthogonal designs without replicated pure-error terms, but the results should be interpreted together with the range analysis rather than as a complete interaction model.

The ANOVA results indicate that GMH-1 dosage (D) and NaOH dosage (C) have statistically significant effects on the comprehensive evaluation index (*p* < 0.05), whereas re-action time (B) shows a marginal effect (*p* = 0.089, 0.05 < *p* < 0.10). Reaction temperature (A) was pooled as the error term and should not be regarded as an independently significant factor. Thus, the order D > C > B > A reflects the trend obtained from range analysis and engineering evaluation, while only D and C were statistically significant under the present ANOVA treatment [15].

### 2.2. Structural and Molecular Characterization

#### 2.2.1. Spectroscopic and Elemental Evidence of Low-Substitution Etherification

**Figure 2:**
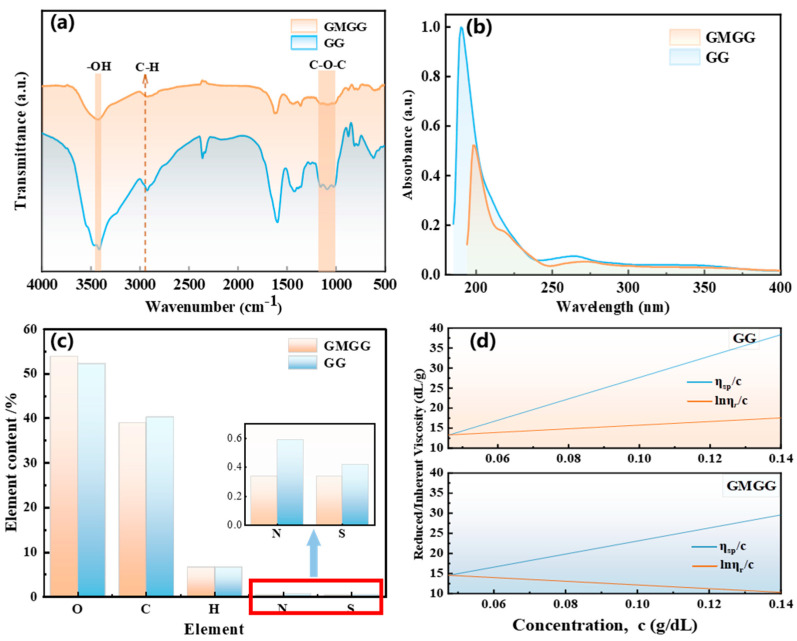
Structural characterization of GG and GMGG: (**a**) FTIR spectra, (**b**) UV-Vis spectra, (**c**) elemental composition, and (**d**) Intrinsic viscosity.

The structural changes in GG after modification with GMH-1 were investigated by FTIR spectroscopy, UV-Vis spectroscopy, and elemental analysis, as shown in Figure 2. These complementary characterization methods were used to evaluate whether chemical modification was likely to have occurred and whether the polysaccharide backbone of GG was largely retained during the modification process. Because GG itself contains abundant hydroxyl and ether groups, these methods provide indirect structural evidence and should not be interpreted as definitive molecular-level proof of a specific substitution site or covalent-bonding pattern.

As shown in Figure 2a, GG and GMGG exhibit similar characteristic FTIR absorption bands. The broad band around 3414 cm^−1^ is assigned to O-H stretching, while the absorption near 2900 cm^−1^ corresponds to C-H stretching of -CH_2_/-CH_3_ groups. The region at 1000–1200 cm^−1^ is associated with C-O-C and C-OH vibrations of the polysaccharide structure. The retention of these characteristic bands suggests that the main galactomannan backbone was largely preserved during modification. Compared with GG, the O-H band of GMGG becomes slightly narrower and weaker, and minor changes are observed in the C-H and C-O/C-O-C regions, suggesting an adjusted hydrogen-bonding environment and changes in the local ether/oxygen-containing chemical environment. However, because native GG already contains abundant hydroxyl and ether linkages, FTIR alone cannot unambiguously prove newly formed ether bonds; the spectral changes are interpreted as being consistent with, rather than definitive proof of, low-substitution etherification [16].

The UV-Vis spectra provide additional evidence for local environmental changes after modification. As shown in Figure 2b, GG and GMGG show similar absorption profiles in the range of 200–400 nm, indicating that the modification process did not cause obvious changes in the overall UV absorption behavior of GG. A slight red shift near 200 nm is observed for GMGG, which may be attributed to changes in the local electronic environment caused by oxygen-containing polar groups. UV-Vis spectroscopy alone cannot provide direct evidence for ether-bond formation [16], but this change is consistent with the FTIR results and supports the occurrence of local polar-environment adjustment after modification.

Elemental analysis further supports the possible introduction of oxygen-containing substituent groups. As shown in Figure 2c, the contents of N and S remain nearly unchanged after modification, indicating that no nitrogen- or sulfur-containing groups were introduced during the reaction. In contrast, the carbon content decreases by approximately 1.25%, whereas the oxygen content increases by approximately 1.65%. This compositional change is consistent with the incorporation of GMH-1-derived oxygen-rich side chains onto GG. Taken together, the FTIR, UV-Vis, and elemental analysis results indirectly support the occurrence of chemical modification and the introduction of oxygen-containing hydrophilic groups while largely preserving the original polysaccharide backbone. More direct structural techniques, such as ^1^H NMR, ^13^C NMR, XPS, or SEC/GPC, will be necessary to further verify the covalent bonding pattern and substitution sites.

#### 2.2.2. Semi-Quantitative Estimation of the Degree of Substitution

Based on the molecular structure of the glycidol-type modifier, which contains one reactive epoxy group, together with the low dosage of GMH-1 used in this study and the limited compositional changes observed from elemental analysis and spectroscopic characterization, the etherification reaction is more reasonably described as a plausible low-level single-point grafting process onto the GG molecular chain. Under such mild reaction conditions, extensive multi-point crosslinking or severe structural alteration of the polysaccharide backbone is considered unlikely, but this interpretation remains indirect. This view is consistent with the FTIR results, in which the characteristic absorption bands of the galactomannan backbone are largely retained after modification.

Elemental analysis showed that, compared with GG, the oxygen content of GMGG increased by approximately 1.65%, whereas the contents of N and S remained nearly un-changed. On the assumption that the oxygen increase mainly originates from grafted GMH-1-derived side chains and that no other oxygen-containing impurities remain after washing, the degree of substitution can be semi-quantitatively estimated from the oxygen mass-fraction increment. This assumption introduces uncertainty and therefore the calculated value should be regarded as an apparent degree of substitution rather than an absolute structural parameter.

According to this semi-quantitative estimation, the apparent degree of substitution of GMGG is approximately 0.09. This value indicates that only a limited proportion of hydroxyl sites on the GG molecular chain may have been substituted under the optimized mild reaction conditions. In other words, the modification likely achieved limited but effective chemical grafting, allowing the introduction of hydrophilic ether side chains while avoiding excessive modification of the original polysaccharide structure. It should be emphasized that this DS value is a semi-quantitative estimate based on elemental analysis and is mainly used to indicate the order of magnitude of the modification degree rather than an absolute degree of substitution. Future NMR-based integration or other quantitative structural analyses are required for a more reliable DS determination.

#### 2.2.3. Molecular Weight Characterization

The intrinsic viscosity [η] of GG and GMGG was determined, and the viscosity-average molecular weight (Mv) was estimated using the Mark–Houwink relationship, as shown in Figure 2d. The [η] of the natural GG sample was 13.0 dL/g, corresponding to an estimated Mv of 1.75 × 10^6^ g/mol. After etherification with GMH-1, the [η] of GMGG increased to 14.3 dL/g, and the corresponding estimated Mv rose to 2.00 × 10^6^ g/mol, representing an apparent relative increase of approximately 14.1%. The detailed measurement conditions and the Mark–Houwink constants are described in Section 4.4.2.

It should be noted that the viscosity method was used mainly to compare relative changes in solution behavior rather than to determine the absolute molecular weight or chain conformation. Etherification may alter chain flexibility, hydration ability, aggregation state, and solution conformation, thereby affecting the relationship between intrinsic viscosity and actual molecular weight. Therefore, the reported Mv values should be regarded as comparative viscosity-average parameters rather than direct evidence of polymer main-chain growth or definitive conformational transformation. Further characterization using DLS or SEC/GPC would be required to quantitatively confirm changes in molecular size distribution and hydrodynamic dimensions.

Combined with the aforementioned FTIR, UV-Vis, and elemental analysis results, the modification process appears to largely preserve the galactomannan backbone of GG without obvious evidence of severe crosslinking or chain scission. The enhanced molecular-chain hydration and adjusted hydrodynamic volume are likely important structural factors contributing to the performance improvement of GMGG. The specific effects of these structural adjustments on the thermal response, rheological behavior, and engineering performance will be further discussed in Section 2.6.

### 2.3. Thermal Behavior Analysis

**Figure 3:**
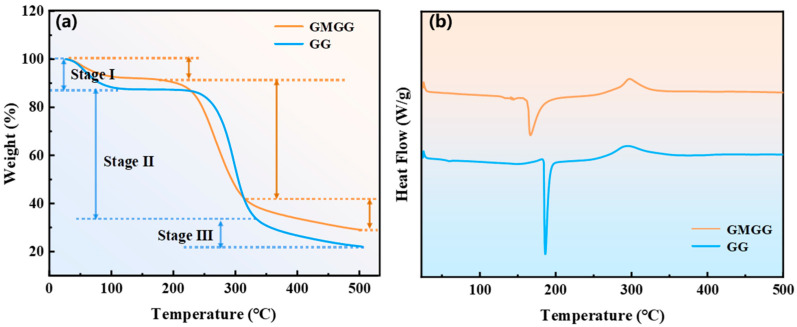
Thermal behavior analysis of GG and GMGG: (**a**) TGA curves; (**b**) DSC curves.

The thermal behavior and stability of GG and GMGG were comprehensively evaluated using TGA and DSC, as shown in Figure 3.

The TGA curves presented in Figure 3a show that both samples undergo a typical multi-stage weight-loss process, indicating certain similarities in their thermal decomposition pathways. Below 150 °C, the weight loss primarily originates from the release of physically adsorbed water. In the 150–300 °C range, the weight-loss rate of GMGG is lower than that of GG, suggesting improved medium-temperature thermal resistance. Further-more, in the 300–500 °C range, the char residue of GMGG is higher, which may be related to modified molecular interactions and altered decomposition behavior [17]. These TGA results suggest that the introduced glycerol ether substituent groups may influence inter-molecular hydrogen bonding, hydration state, or chain flexibility, thereby contributing to the observed thermal-response changes.

The DSC analysis illustrated in Figure 3b further indicates changes in the thermal response after modification. Both samples exhibit thermal transition processes, and the main thermal response of GMGG shifts slightly upward with a gentler heat-flow curve compared with GG. This result is consistent with an adjusted molecular interaction environment after low-substitution modification. However, without coupled techniques such as TG-FTIR or TG-MS, the DSC events cannot be assigned unambiguously to a specific structural decomposition process. Therefore, the DSC data are discussed here as supportive evidence for altered thermal behavior rather than direct proof of a defined decomposition mechanism.

Overall, the TGA and DSC results suggest that GMGG exhibits improved thermal-response behavior in the medium-high temperature range [2,18]. This improvement is favorable for maintaining fracturing-fluid performance, but the molecular origin of the thermal response should be regarded as a plausible interpretation that requires further verification. The specific structure-thermal behavior relationship will be discussed cautiously in Section 2.6.

### 2.4. Rheological Behavior of GMGG Crosslinked Gels

#### 2.4.1. Steady-State and Dynamic Viscoelastic Response

**Figure 4:**
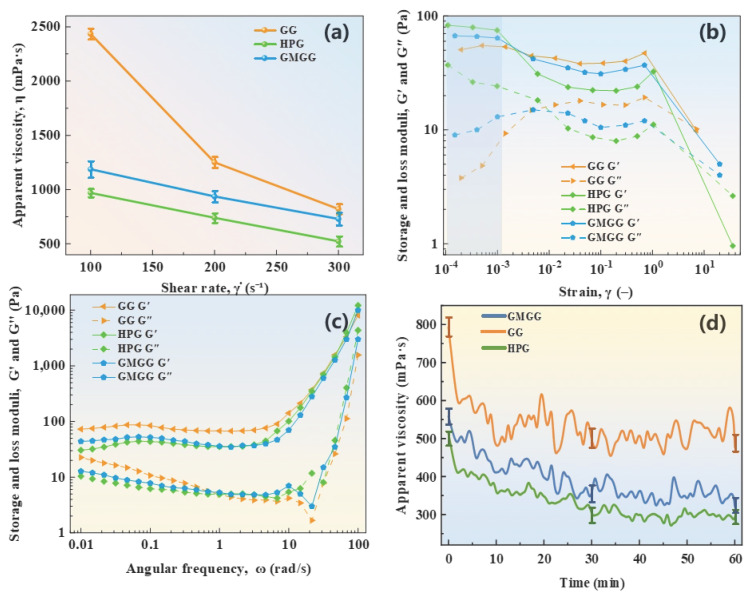
Rheological properties of the crosslinked gels: (**a**) steady-state shear viscosity; (**b**) strain sweep; (**c**) frequency-dependent viscoelastic moduli; (**d**) long-term shear stability.

To evaluate the rheological properties under simulated pumping and prop-pant-carrying conditions, crosslinked gel samples were prepared using 0.6 wt % GMGG, with natural GG and commercial HPG serving as control groups. Before crosslinking, the apparent viscosity of the GMGG linear gel was approximately 65 mPa·s at 100 s^−1^, satisfying the minimum requirement of 30 mPa·s specified by the SY/T 5764-2007 standard. Following borate crosslinking, the steady flow behaviors recorded at 60 °C are presented in Figure 4a. All three crosslinked systems exhibit typical shear-thinning pseudoplastic behavior [19,20,21], characterized by a continuous decrease in viscosity as the shear rate increases from 10 to 300 s^−1^. At the critical shear rate of 100 s^−1^, the apparent viscosity of the GMGG crosslinked gel reaches approximately 1170 mPa·s. This indicates the formation of a strong three-dimensional network and suggests good proppant-carrying potential under the tested conditions [22].

The structural integrity of these gels was further investigated through strain and frequency sweeps to define the linear viscoelastic region (LVR). As shown in Figure 4b, GMGG maintains a stable storage modulus (G’) up to a strain of 3.4%, comparable to the 3.7% observed for commercial HPG and wider than the 1.2% measured for natural GG. This indicates that the GMGG gel network has improved resistance to mechanical disturbance [23]. Furthermore, frequency scanning from 0.1 to 100 rad/s in Figure 4c shows elasticity-dominated behavior of GMGG, with G’ remaining higher than G″ across most of the tested range. Compared with GG, GMGG exhibits modulus stability closer to that of HPG, suggesting a more continuous and persistent crosslinked network structure [20,24].

#### 2.4.2. Long-Term Shear Stability and Structure Retention

The dynamic stability of the gels was assessed under continuous shear at 170 s^−1^ for 60 min at 60 °C, as shown in Figure 4d. Natural GG exhibited rapid viscosity loss over time, whereas GMGG showed a more controlled decrease. According to the original rheo-logical curve, the apparent viscosity of GMGG was approximately 558.5 mPa·s at the beginning of the test, approximately 413.0 mPa·s at 30 min, and approximately 329.9 mPa·s at 60 min, corresponding to a 30 min viscosity retention ratio of about 73.9%. This value exceeds the 70% retention requirement specified by SY/T 5107-1995. These results suggest that the crosslinked GMGG network can maintain acceptable shear resistance under the tested conditions, although further high-temperature and high-pressure rheological tests are still needed for field-scale validation [2,25].

Error bars in Figure 4a represent the standard deviations of three independent measurements. For Figure 4d, the continuous curves represent representative viscosity evolution during long-term shear, while error bars are shown at selected characteristic time points (0, 30, and 60 min) for clarity and represent the standard deviations of three independent measurements. The strain and frequency sweep curves in Figure 4b,c are presented as representative dynamic rheological profiles.

### 2.5. Evaluation of Engineering Application Performance

#### 2.5.1. Filtration Control and Clay Swelling Inhibition

The interaction between the fracturing fluid and the reservoir was first evaluated through filtration and clay swelling tests to assess fluid retention and formation stability. Filtration behaviors were compared across a temperature range of 40, 60, and 80 °C, as detailed in Table 4.

The results show that GMGG exhibits relatively low filtration volume and rate among the three systems. This behavior is particularly evident at 80 °C, where GMGG records a filtration rate of 4.01 × 10^−5^ m/min. This performance meets the SY/T 5107-1995 criteria, which require an initial loss of 2.0 × 10^−3^ m^3^·m^−2^ or less and a filtration coefficient of 4.0 × 10^−4^ m·min^−1/2^ or less. These findings indicate that the modified system has good fluid-retention capacity under the tested conditions and may help reduce fluid invasion into the formation [26].

**Figure 5:**
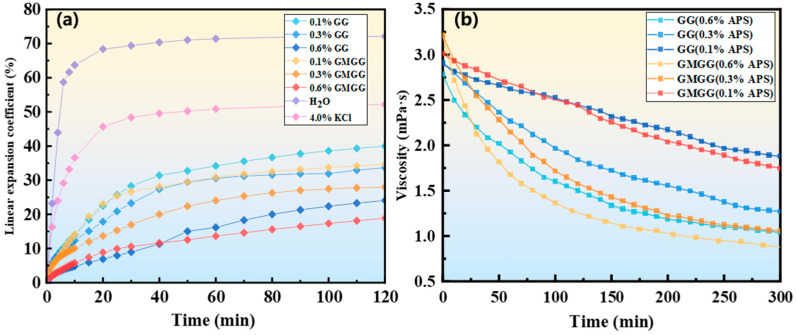
Evaluation of engineering application performance: (**a**) Clay swelling inhibition; (**b**) Effect of breaker dosage on gel-breaking.

In addition to filtration control, GMGG shows a capacity to retard the hydration and swelling of sodium bentonite, as illustrated in Figure 5a. In the natural GG system at 0.6 wt %, the linear expansion coefficient of bentonite increases continuously and reaches approximately 24% after 120 min. In contrast, the GMGG system tends to stabilize after about 30 min and is finally controlled at approximately 19%. Compared with the expansion levels of approximately 72% in deionized water and 52% in 4.0 wt % KCl solution, the modified thickener reduces clay swelling under the tested conditions. This inhibition is likely associated with the higher solution viscosity, reduced free-water migration, and possible formation of a polymer hydration or adsorption layer on clay surfaces, rather than a direct ion-exchange mechanism like that of KCl [27]. Therefore, the GMGG system may be suitable for fracturing operations in reservoirs containing water-sensitive minerals, although additional clay-mineral-specific tests are required.

#### 2.5.2. Gel-Breaking Performance and Reservoir Damage Assessment

The post-treatment performance of the fracturing fluid, including degradability and subsequent impact on reservoir permeability, was investigated. Gel-breaking tests using ammonium persulfate (APS) show a dose-dependent degradation process, as shown in Figure 5b. Low APS dosages of 0.1% result in incomplete breaking for both systems, whereas a dosage of 0.6% allows GMGG to reach a low apparent viscosity of approximately 1.0 mPa·s within 2 h and largely eliminates its viscoelasticity [28]. This response meets the SY/T 6376-2011 requirement for low-residue flowback, suggesting that the fluid can be more easily removed after stimulation [29].

The effectiveness of this clean-breaking behavior is further reflected in the static core damage evaluation presented in Table 5. Sandstone cores treated with a 0.5% GMGG solution exhibited permeability damage rates ranging from 29.45% to 31.03%. These values fall within the commonly accepted tolerance range of 20% to 40% for fracturing fluids [30]. Collectively, the residue testing, rheological stability, and core damage data suggest that the GMGG system provides a favorable balance between thickening efficiency and reservoir protection under the laboratory conditions used in this study [3,4,31].

### 2.6. Analysis of Modification Mechanism and Structure-Property Relationship

#### 2.6.1. Explanation of Reaction Path of Etherification Modification

**Figure 6:**
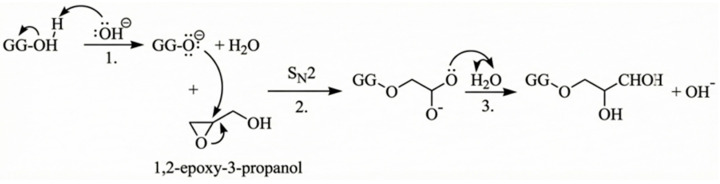
Proposed etherification reaction pathway of GG with GMH-1 under alkaline conditions.

Under alkaline conditions, the hydroxyl groups on the GG molecular chain can be deprotonated and activated, enabling reaction with epoxy compounds. When the glycerol ether modifier GMH-1 is introduced, the activated hydroxyl groups may initiate the ring-opening reaction of epoxy groups through nucleophilic attack, leading to a side-chain structure connected by ether linkages on the GG molecular chain. This reaction route is consistent with the general etherification behavior of epoxy compounds under alkaline conditions, and a plausible schematic pathway is shown in Figure 6 [32].

It should be pointed out that Figure 6 is intended to illustrate a plausible reaction pathway rather than to provide direct proof of the exact reaction mechanism. The present FTIR, UV-Vis, and elemental analysis results support chemical modification indirectly, but they do not determine the precise substitution site, the local bonding environment, or the distribution of substituent groups along the GG chain. Based on this evidence level, the following discussion focuses on reasonable structure-property interpretations relevant to engineering performance.

#### 2.6.2. Influence of Structural Changes on Hydration Behavior and Residue Reduction

FTIR spectroscopy, UV-Vis spectroscopy, and elemental analysis collectively suggest that oxygen-containing hydrophilic side groups may have been introduced while the polysaccharide backbone of GG was largely retained. Such structural adjustment may enhance interactions between polymer chains and water molecules, thereby improving hydration and swelling behavior and making the molecular chains more likely to expand and disperse in aqueous solution.

From the perspective of solution conformation, the introduced hydrophilic side groups may increase the interaction strength between molecular chains and water molecules and alter the expansion mode of GG molecules in solution, leading to a larger effective hydrodynamic volume. This conformational change is likely conducive to more uniform molecular dispersion, which can indirectly improve hydration behavior and positively affect macroscopic rheological properties.

On this basis, the reduction in water-insoluble residue content after modification can be reasonably associated with improved hydration and dispersion behavior. A more sufficient and uniform molecular hydration state may reduce the formation or persistence of insufficiently hydrated particles or insoluble aggregates during swelling and dispersion, resulting in a lower proportion of water-insoluble matter in the residue test.

It should be emphasized that this study has not directly characterized the particle size distribution, morphology, or dissolution kinetics of residue particles. Therefore, the above explanation is based on macroscopic residue tests and indirect structural evidence, and should be regarded as a reasonable mechanistic inference rather than a quantitative proof of the residue reduction mechanism. Future SEM, particle-size analysis, and dissolution-kinetics measurements would be valuable for verifying this interpretation.

#### 2.6.3. Relationship Between Single-Point Grafting Characteristics and Rheological Performance Stability

Since the GMH-1 molecule contains one reactive epoxy group and the modifier dos-age is low in this study, the etherification reaction is more likely to occur through low-level single-point grafting rather than extensive multi-point crosslinking or main-chain scission. This interpretation is consistent with the retention of the major polysaccharide FTIR bands and the absence of abnormal gelation behavior during solution preparation, but it should be regarded as an inference based on indirect evidence.

Retention of the main-chain structure is beneficial for maintaining the inherent rheo-logical properties and processability of the GG system. While maintaining pseudoplastic flow characteristics, the modified system shows good viscoelastic stability and shear tolerance. These improvements are more likely related to changes in hydrodynamic volume, hydration, and solution conformation than to a true increase in main-chain length or formation of permanent crosslinked structures.

#### 2.6.4. Correlation Between Structural Homogeneity and Crosslinked Gel Network Performance

In the crosslinked gel system, the dissolution and dispersion state of the polymer solution has an important influence on the formation of the three-dimensional network structure. A more uniform and sufficient molecular dispersion state is conducive to the formation of a network system with better structural continuity during crosslinking, thus reducing the generation of local structural defects.

The reduction of such structural defects, rather than the simple increase of crosslinking density, is considered to help improve the overall structural stability of crosslinked gels under shear and oscillation conditions. This structure-property correlation provides a reasonable explanation for the comprehensive performance of GMGG in shear stability, viscoelastic properties and filtration control [32,33].

#### 2.6.5. Engineering Significance and Applicable Scope of Mechanism Analysis

From the perspective of engineering application, the reduction in water-insoluble residue content and the improvement of rheological structure stability may help reduce the risk of adverse effects of solid residues in the fracturing fluid system on fracture and pore structures. However, reservoir damage is the result of the coupling of multiple factors, and residue content is only one of the influencing factors.

Overall, low-substitution glycerol etherification appears to provide a useful balance between hydrophilicity enhancement and residue reduction without substantially altering the natural galactomannan backbone. This molecular design offers a rational framework for improving guar-based fracturing fluid thickeners, but the industrial potential of GMGG should be further validated through more direct structural characterization, dynamic core-flow experiments, and field-relevant high-temperature/high-pressure tests.

Although the present results demonstrate the potential of GMGG under laboratory conditions, its practical applicability under realistic reservoir conditions still requires further verification. In particular, high-salinity brines, elevated temperatures beyond the current test range, long-term thermal aging, and dynamic core flooding experiments should be considered in future work. These tests will be essential for evaluating polymer stability, residue transport, permeability recovery, and formation damage under field-relevant conditions.

Compared with conventional HPG production, the proposed low-substitution glycerol etherification route may offer several potential advantages. First, the low modifier dosage and mild reaction conditions may reduce chemical consumption and processing severity. Second, the glycerol ether-based modification strategy may reduce reliance on volatile propylene oxide commonly associated with traditional hydroxypropylation. Third, the reduced insoluble residue content may help decrease formation damage and improve flowback cleanliness. Nevertheless, a complete techno-economic and life-cycle assessment was not conducted in this study, and further evaluation is required before industrial implementation.

## 3. Conclusions

In this study, a mild glycerol etherification strategy was developed to prepare a reduced-residue guar gum-based fracturing fluid thickener (GMGG). Under the optimized conditions of 35 °C, 2.0 h, 2.0 wt % NaOH, and 0.2 wt % GMH-1, the water-insoluble residue content of GMGG decreased to 5.1% compared with 12.4% for natural GG, while the apparent viscosity of its 0.6 wt % solution increased to 125.3 mPa·s. FTIR, UV-Vis spectroscopy, elemental analysis, and intrinsic-viscosity measurements collectively suggested that low-level chemical modification occurred and that oxygen-containing hydrophilic side groups were introduced while the main galactomannan backbone was largely retained. Because these techniques provide indirect structural evidence, the apparent degree of substitution estimated in this work should be regarded as a semi-quantitative indicator of the modification level rather than an absolute structural parameter. Rheological, thermal, filtration, gel-breaking, clay-swelling, and core-damage evaluations further indicated that GMGG has potential as a reduced-residue thickener for water-based fracturing fluids. The improved performance is likely associated with enhanced hydration, dispersion, and crosslinked-network continuity, which may reduce the formation or persistence of insufficiently hydrated aggregates. Nevertheless, the proposed reaction pathway and residue-reduction mechanism remain plausible interpretations based on indirect evidence. Future work should include ^1^H NMR, ^13^C NMR, XPS, SEC/GPC, residue particle-size and morphology analyses, and pilot-scale core displacement tests to verify the substitution pattern, molecular architecture, and field applicability of GMGG more quantitatively.

## 4. Materials and Methods

### 4.1. Materials

Natural guar gum (GG) powder was provided by Shaanxi Yanchang Petroleum (Group) Co., Ltd. (Xi’an, China). The glycerol ether modifier GMH-1 was obtained from Shandong Guangmao Petroleum Technology Service Co., Ltd. (Dongying, China). Sodium hydroxide, glacial acetic acid, anhydrous ethanol, ammonium persulfate, and sodium tetraborate were of analytical grade and purchased from Sinopharm Chemical Reagent Co., Ltd. (Shanghai, China). Deionized water was used throughout all experiments.

### 4.2. Preparation of GMH-1 Modified Guar Gum

GG powder was first dispersed in a mixed solvent of deionized water and anhydrous ethanol. A predetermined amount of NaOH was then added under continuous stirring to activate the hydroxyl groups of GG under alkaline conditions. Subsequently, the glycerol ether modifier GMH-1 was introduced, and the etherification reaction was carried out at a specified temperature for a defined period.

After the reaction, the system was adjusted to near neutrality (pH ≈ 7) using glacial acetic acid. The product was collected by vacuum filtration and washed several times with anhydrous ethanol to remove unreacted reagents and physically extractable impurities. The resulting material was then dried at room temperature to obtain the GMGG, denoted as GMGG.

A blank control was also prepared by subjecting natural GG to the same heat treatment and ethanol-washing procedure in the absence of GMH-1. This sample was used to distinguish the contribution of chemical modification from that of physical purification in the subsequent analyses.

### 4.3. Orthogonal Experimental Design

In the process of etherification modification of GG with GMH-1, reaction conditions have a significant impact on modification efficiency and product performance. Based on previous single-factor experiments, four main factors, including reaction temperature (A), reaction time (B), NaOH dosage (C), and GMH-1 dosage (D), were selected, and the L9(3^4^) orthogonal table was used for optimization. This design was used mainly for main-effect screening; interaction effects among factors were not included in the statistical model. The factors and levels are shown in Table 6.

Considering the requirements of engineering application, the apparent viscosity and water-insoluble residue content of 0.6 wt % aqueous solution were selected as performance evaluation indicators. All nine orthogonal experimental products were tested, and the optimized product was then used for detailed characterization and application-performance evaluation. Because the two indicators have different units and optimization directions, the extreme-value normalization method was used, and a comprehensive evaluation index (CI) was constructed with equal weights for orthogonal analysis and subsequent statistics.

Specifically, for the apparent viscosity V, where a higher value is desired, the normalized value $V_{norm}$ was calculated as:

$$V_{norm}=\frac{V_{i}-V_{min}}{V_{max}-V_{min}}$$

For the insoluble residue content R, where a lower value is desired, the normalized value $R_{norm}$ was calculated as:

$$R_{norm}=\frac{R_{max}-R_{i}}{R_{max}-R_{min}}$$

The comprehensive evaluation index (CI) was then expressed as a percentage:

$$CI=(0.5\times V_{norm}+0.5\times R_{norm})\times 100$$

In addition, all test data are average values from at least three parallel measurements unless otherwise specified. The L9 design was used for efficient optimization and main-effect analysis, and more detailed interaction analysis will require replicated response-surface or full-factorial experiments.

### 4.4. Characterization and Performance Evaluation

#### 4.4.1. Structural Characterization

The molecular structures and functional groups of GG and GMGG were systematically characterized. Fourier-transform infrared (FTIR) spectra were recorded using a Nicolet 5700 spectrometer (Thermo Fisher Scientific, Waltham, MA, USA) via the solid KBr pellet method in the wavenumber range of 4000–400 cm^−1^. For UV-Vis analysis, 0.08 wt % aqueous solutions of GG and GMGG were prepared and allowed to fully swell. The solutions were then placed in a sample cell, and UV-Vis spectra were recorded using a UV-2600 UV–Vis spectrophotometer (Shimadzu Corporation, Kyoto, Japan) in the wavelength range of 185–400 nm to evaluate changes in the molecular electronic environment and polar characteristics before and after modification. A vario EL cube elemental analyzer (Elementar Analysensysteme GmbH, Langenselbold, Germany) was used to determine the contents of C, H, N, S, and O in GG and GMGG. These elemental analysis results were used to compare overall chemical composition changes and to perform a semi-quantitative estimation of the apparent degree of substitution. It should be noted that FTIR, UV-Vis, and elemental analysis provide indirect evidence of chemical modification; they cannot determine the exact covalent-bonding pattern, substitution position, or complete molecular architecture.

#### 4.4.2. Molecular Weight Determination

The relative viscosity of diluted GG and GMGG solutions was measured with an Ubbelohde viscometer at 25 °C. A series of dilute polymer solutions was prepared, and the intrinsic viscosity [η] was obtained by extrapolating the reduced and inherent viscosity values to zero concentration. Each viscosity measurement was performed in triplicate, and the viscosity-average molecular weight was estimated according to the Mark–Houwink equation:

$$\left[\eta \right]=KM_{v}^{\alpha }$$

The molecular weight was then calculated as $M_{v}={\left(\left[\eta \right]/K\right)}^{1/\alpha }$ with a fitting correlation coefficient $R^{2}>0.99$, where [η] is the intrinsic viscosity (dL/g) determined in Section 2.2.3. The empirical constants K and α for the GG aqueous solution at 25 °C were taken as 3.8 × 10^−4^ dL/g and 0.723, respectively, based on the established literature for guar galactomannan systems [34]. The calculated Mv values were used only as comparative viscosity-average parameters reflecting changes in hydrodynamic volume and solution conformation. Accurate absolute molecular-weight distribution would require SEC/GPC analysis, which is beyond the scope of the present revision.

#### 4.4.3. Thermal Analysis

To investigate the thermal response and decomposition behavior of the samples be-fore and after modification, thermogravimetric analysis (TGA) and differential scanning calorimetry (DSC) were used. TGA tests were carried out under nitrogen (20 mL/min) and air (50 mL/min) atmospheres with a heating rate of 10 °C/min over 25–500 °C. DSC tests were performed with a Mettler Toledo DSC-822e instrument, and the endothermic and exothermic processes were recorded under the same temperature program. Because no coupled TG-FTIR or TG-MS analysis was performed, DSC events were interpreted only as changes in thermal response behavior rather than as definitive evidence for specific de-composition reactions.

#### 4.4.4. Performance Evaluation

The engineering performance of GG and GMGG was evaluated based on relevant industry standards, including SY/T 5764-2007 for viscosity and residue, SY/T 5107-1995 for temperature stability, shear stability, and filtration performance, and SY/T 6335-1997 for clay swelling inhibition. Gel-breaking behavior was assessed using ammonium persulfate as the breaker.

## Figures and Tables

**Table 1:** Results of orthogonal experiments and comprehensive evaluation index.

Run	A	B	C	D	Apparent Viscosity (mPa·s)	Insoluble Residue (%)	Comprehensive Index
1	1	1	1	1	97.99	10.82	0.00
2	1	2	2	2	128.56	4.99	100.00
3	1	3	3	3	126.26	5.12	95.30
4	2	1	2	3	123.74	5.35	89.21
5	2	2	3	1	117.31	9.21	45.40
6	2	3	1	2	123.99	6.41	80.38
7	3	1	3	2	124.11	5.83	85.62
8	3	2	1	3	117.36	8.56	50.99
9	3	3	2	1	120.73	7.75	63.58

**Table 2:** Range analysis results of the orthogonal experiment.

Level	A	B	C	D
K_1_	65.10	58.28	43.79	36.33
K_2_	71.66	65.46	84.26	88.67
K_3_	66.73	79.75	75.44	78.50
R	6.56	21.47	40.47	52.34
Variance	D > C > B > A			
Optimal combination	D_2_C_2_B_3_A_2_			

**Table 3:** Analysis of variance (ANOVA) results for the orthogonal experiment.

Factor	Sum of Squares (SS)	Degree of Freedom (df)	Mean Square (MS)	F Value	*p* Value
B	717.10	2	358.55	10.23	0.089
C	2717.66	2	1358.83	38.78	0.025
D	4621.43	2	2310.71	65.95	0.015
A	70.07	2	35.04	-	-
Total	8126.26	8			

**Table 4:** Filtration Performance at Various Temperatures.

Temperature (°C)	Sample	Initial Filtration Loss (m^3^·m^−2^)	Filtration Coefficient (m·min^−1/2^)	Filtration Rate (m·min^−1^)
40.0	GG	1.10 × 10^−3^	1.41 × 10^−4^	2.35 × 10^−5^
	GMGG	0.80 × 10^−3^	1.15 × 10^−4^	1.92 × 10^−5^
	HPG	1.05 × 10^−3^	2.20 × 10^−4^	3.85 × 10^−5^
60.0	GG	1.26 × 10^−3^	2.91 × 10^−4^	4.85 × 10^−5^
	GMGG	1.10 × 10^−3^	2.73 × 10^−4^	4.55 × 10^−5^
	HPG	1.20 × 10^−3^	2.73 × 10^−4^	4.80 × 10^−5^
80.0	GG	1.41 × 10^−3^	3.54 × 10^−4^	5.90 × 10^−5^
	GMGG	1.36 × 10^−3^	2.43 × 10^−4^	4.01 × 10^−5^
	HPG	1.35 × 10^−3^	3.30 × 10^−4^	5.35 × 10^−5^

**Table 5:** Static Core Damage Evaluation of the GMGG Fracturing Fluid.

Number	Diameter (cm)	Length (cm)	K_0_ (mD)	K_1_ (mD)	D (%)
1	2.58	5.79	20.54	14.49	29.45
2	2.55	5.45	19.27	13.29	31.03
3	2.53	6.13	27.38	19.09	30.28

Note: K_0_: Initial permeability; K_1_: Post-treatment permeability; D: Damage rate.

**Table 6:** Factors and levels of the orthogonal experimental design.

Level	A: Temperature (°C)	B: Time (h)	C: NaOH Dosage (wt % of GG)	D: GMH-1 Dosage (wt % of GG)
1	30	1.0	1.5	0.15
2	35	1.5	2.0	0.20
3	40	2.0	2.5	0.25

## Data Availability

The original contributions presented in the study are included in the article, further inquiries can be directed to the corresponding authors.

## Abbreviations

The following abbreviations are used in this manuscript:

GG	Natural guar gum
GMGG	Modified product
GMH-1	Glycerol ether-based modifier
HPG	Hydroxypropyl guar gum
